# New Journal

**Published:** 1869-06-15

**Authors:** 


### *New Journal.*

We note, with pleasure, the announcement of *The Journal of the Gynæcological Society of Boston*, under the management of its officers, Drs. Winslow Lewis, H. R. Storer and George H. Bixby. The first number will be issued July 1st, and be continued monthly. $3.00 *per annum* or 35 cts. per number. JAMES CAMPBELL, 18 Tremont Street Boston, Publisher.

